# The Neutrophil Percentage-to-Albumin Ratio Is Associated with All-Cause Mortality in Critically Ill Patients with Acute Kidney Injury

**DOI:** 10.1155/2020/5687672

**Published:** 2020-02-18

**Authors:** Benji Wang, Diwen Li, Bihuan Cheng, Binyu Ying, Yuqiang Gong

**Affiliations:** Department of Anesthesiology, Critical Care and Pain Medicine, The Second Affiliated Hospital and Yuying Children's Hospital of Wenzhou Medical University, Wenzhou 325000, Zhejiang, China

## Abstract

**Background:**

There is no evidence to suggest the predictive power of neutrophil percentage-to-albumin ratio (NPAR) in patients with acute kidney injury (AKI). We hypothesized that NPAR would correlate with all-cause mortality in critically ill patients with AKI.

**Methods:**

From the MIMIC-III V1.4 database, we extracted demographics, vital signs, comorbidities, laboratory tests, and other clinical data. The clinical endpoints were 30-, 90- and 365-day all-cause mortality in critically ill patients with AKI. Cox proportional hazards models were used to evaluate the prognostic values of NPAR, and subgroup analyses were performed to measure mortality across various subgroups.

**Results:**

A total of 7,481 eligible subjects were enrolled. In multivariate analysis, after adjustments for age, ethnicity, gender, and other confounding factors, higher NPARs were associated with an increased risk of 30-, 90- and 365-day all-cause mortality in critically ill patients with AKI (tertile 3 versus tertile 1: adjusted HR, 95% CI: 1.48, 1.30–1.69; 1.47, 1.31–1.66; 1.46, 1.32–1.62, respectively; *P* trend <0.01). A similar trend was observed in the NPAR group division by quintiles. Subgroup analysis revealed no significant interactions in most strata.

**Conclusions:**

Increased NPAR correlates with increased risk of all-cause mortality in critically ill patients with AKI.

## 1. Introduction

Acute kidney injury (AKI) is defined as a sudden deterioration of renal function and is associated with high morbidity and mortality [1], especially for critically ill patients. In the US, 6–24% of critically ill patients in intensive care units (ICUs) have AKI [2] and the mortality rate of these patients is as high as 60–70% [3], greatly increasing healthcare costs and imposing substantial healthcare burdens. Given the poor prognosis of AKI in critical illness, finding novel biomarkers to identify the severity of AKI and adopting early effective interventions to improve survival are critical. Investigators have sought several few biomarkers of mortality in AKI [4, 5]; nevertheless, these efforts have been largely unsuccessful.

The pathogenesis of AKI has not been fully elucidated; progression of AKI may be associated with systemic inflammatory [6–8]. Leukocytes, including neutrophils, produce inflammatory mediators such as cytokines and chemokines that damage the kidneys. Albumin is a crucial protein with several functions, including osmotic pressure regulation and antioxidant and anti-inflammatory effects [9, 10]; it too has been associated with AKI [11]. Therefore, we hypothesize that neutrophil percentage-to-albumin (NPAR) could serve as an inflammation-based prognostic score. Neutrophil-to-albumin ratio (NAR) has been identified as a biomarker predicting prognosis in patients with rectal cancer and end-stage pancreatic cancer [12, 13]. Based on these findings, we have reason to speculate that NPAR may affect the prognosis of AKI in critical illness. To our knowledge, there has been no epidemiological study exploring the association between NPAR and mortality in critically ill patients with AKI.

## 2. Methods

### 2.1. Data Source

Similar to our previous studies, we followed the methods of Wang et al. [14, 15]. The Multiparameter Intelligent Monitoring in Intensive Care III version 1.4 (MIMIC-III v1.4) is an openly available dataset. It includes deidentified health data derived from ∼40,000 critical care patients from 2001 to 2012 [16]. To apply for access to the database, we passed the Protecting Human Research Participants exam and obtained a certificate (No. 6182750). The project is approved by the institutional review boards of the Massachusetts Institute of Technology and Beth Israel Deaconess Medical Center and was granted a waiver of informed consent.

### 2.2. Population Selection Criteria

We restricted the search to adult patients (≥18 years) with AKI. The occurrence of AKI was determined on the basis of Kidney Disease: Improving Global Outcomes (KDIGO) definition [17], and Structured Query Language (SQL) for extracting AKI was included in supplementary material. For inclusion, patients needed to be hospitalized in the ICU at first admission for more than two days. Patients who met the following criteria were excluded: (1) no neutrophil percentage and albumin measured during ICU stay and (2) more than 5% of individual data missing.

### 2.3. Data Extraction

SQL with PostgreSQL tools (version 9.6) was used to extract the data from MIMIC-III. Demographics, vital signs, comorbidities, laboratory tests, and others were extracted. The comorbidities included coronary artery disease (CAD), congestive heart failure (CHF), atrial fibrillation (AFIB), stroke, renal disease, liver disease, pneumonia, malignancy, and respiratory failure. Laboratory tests included neutrophil percentage, albumin, bicarbonate, creatinine, chloride, glucose, hematocrit, hemoglobin, platelet, sodium, potassium, blood urea nitrogen (BUN), white blood cell (WBC), prothrombin time (PT), activated partial thromboplastin time (APTT), and international normalized ratio (INR). Sequential organ failure assessment (SOFA) score [18] and simplified acute physiology score II (SAPSII) [19] were calculated for each patient at the time of ICU admission. The other extracted data included age, gender, ethnicity, systolic blood pressure (SBP), diastolic blood pressure (DBP), mean blood pressure (MBP), heart rate, respiratory rate, temperature, SPO2, AKI stage, renal replacement therapy, vasopressor use, and length of stay in the ICU. Records containing laboratory tests were extracted within 24 hours after admission to the ICU. Survival information regarding vital status was obtained from the Social Security Death Index records. The endpoints for this analysis were 30-day, 90-day, and 365-day all-cause mortality.

### 2.4. Statistical Analysis

Baseline characteristics of all patients were stratified by NPAR tertiles. Continuous variables were presented as mean ± standard deviation (SD), and categorical data were summarized as number or percentage. We used chi-square or one-way ANOVA to test for differences in categorical or continuous factors among various categories of NPAR. The prognostic values of NPAR were evaluated using Cox proportional hazards models, and the results were presented as hazard ratios (HRs) with 95% confidence intervals (CIs).

Two multivariate models were constructed on the basis of NPAR group inclusion according to tertiles and quintiles based on 30-, 90-, and 365-day all-cause mortality. The first tertile or quintile was treated as the reference group. In model I, covariates were only adjusted for age, ethnicity, and gender. In model II, we further adjusted for age, ethnicity, gender, AKI stage, CHF, AFIB, liver disease, CAD, stroke, malignancy, respiratory failure, pneumonia, sodium, potassium, chloride, BUN, INR, APTT, platelet, WBC, hematocrit, creatinine, glucose, bicarbonate, vasopressor use, heart rate, SBP, DBP, respiration rate, temperature, SPO2, SOFA, SAPSII, and renal replacement therapy. We selected these confounders based on a change in effect estimate of more than 10% [20]. Subgroup analyses were performed to evaluate whether the effect of the 30-day mortality differed across various subgroups classified by CHF, AFIB, CAD, stroke, malignancy, liver disease, respiratory failure, pneumonia, AKI stage, WBC, sodium, BUN, INR, potassium, APTT, platelet, hematocrit, creatinine, bicarbonate, glucose, chloride, SBP, DBP, heart rate, respiratory rate, temperature, SPO2, SOFA score, SAPSII score, vasopressor use, and renal replacement therapy.

Receiver-operating characteristic (ROC) curve was performed to measure the sensitivity and specificity of NPAR, neutrophils percentage, albumin, and SOFA score. Moreover, the area under the curve (AUC) was calculated to evaluate the quality of NPAR as a predictor of 30-day all-cause mortality. All statistical analyses were performed using EmpowerStats version 2.17.8 (http://www.empowerstats.com/cn/, X&Y solutions, Inc., Boston, MA) and *R* software version 3.42; *P* < 0.05 was considered statistically significant.

## 3. Results

### 3.1. Subject Characteristics

**Figure 1:**
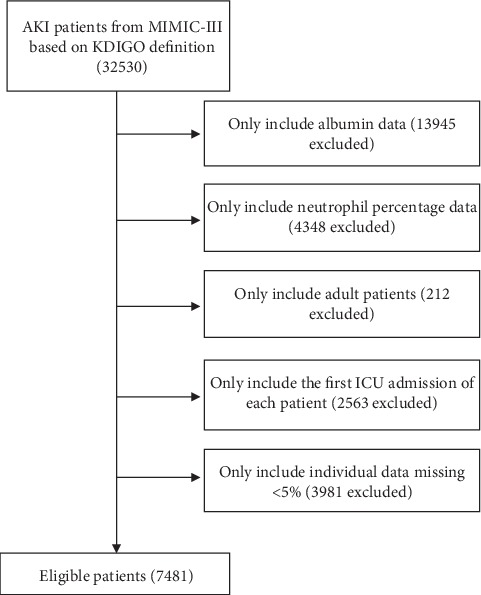
Illustration of exclusion and inclusion criteria as utilized to select the final 7481 patients.

A total of 7,481 eligible subjects were enrolled (Figure 1). Characteristics of the study patients stratified by NPARs tertiles are displayed in Table 1. A total of 2,492 patients were in the low-NPAR group (tertile 1: NPAR < 22.1), 2,494 patients were in the mid-NPAR group (tertile 2: 22.1–28.1), and 2,495 patients were in the high-NPAR group (tertile 3: NPAR ≥ 28.1). The subjects included 3,245 women and 4,236 men, most of whom were white. Patients with high NPAR values (NPAR ≥ 28.1) were more likely to receive renal replacement therapy and vasopressors and to report a history of stroke, liver disease, malignancy, and respiratory failure; they also had lower SBP, DBP, MBP, bicarbonate, hematocrit, and hemoglobin; finally, they also had higher levels of heart rate, respiratory rate, creatinine, chloride, platelet, BUN, WBC, PT, APTT, SOFA, SAPSII, ICU LOS, and mortality.

### 3.2. NPAR as a Predictor of the Clinical Endpoints

**Figure 2:**
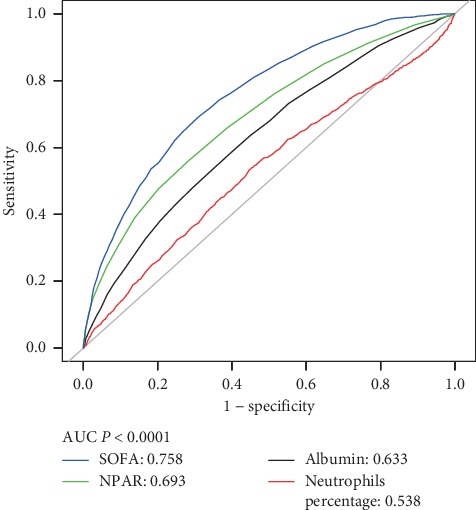
ROC curves for the prediction of 30-day all-cause mortality in critically ill patients with AKI. AUCs for NPAR, neutrophils percentage, albumin, and SOFA score were 0.693, 0.538, 0.633, and 0.758.

In model I, after adjustments for age, ethnicity, and gender, higher NPARs were associated with increased risk of all-cause mortality than were the first tertile (<22.1) or quintile (<19.6). In model II, after adjusting for more confounding factors, NPAR was also an independent predictor of 30-, 90-, and 365-day all-cause mortality in critically ill patients with AKI (tertile 3 versus tertile 1: adjusted HR, 95% CI: 1.48, 1.30–1.69; 1.47, 1.31–1.66; 1.46, 1.32–1.62, *P* trend <0.01). A similar trend was observed in NPAR group inclusion according to quintiles (Table 2). Moreover, the ROC curves were generated, and we found that the AUCs for NPAR, neutrophils percentage, albumin, and SOFA score were 0.693, 0.538, 0.633, and 0.758, respectively (Figure 2). Comparing AUCs, NPAR was found to be lower than SOFA score but was a better predictor than neutrophil percentage or albumin alone (*P* < 0.01).

### 3.3. Subgroup Analyses

There were no significant interactions in most strata in the subgroup analyses (Table 3). Patients with high values of potassium, platelet, hematocrit, bicarbonate, and SBP had higher risks of all-cause mortality for high NPAR. Similarly, patients with SOFA scores <4, SAPSII scores <34, temperature <36.5°C, heart rate <80 beats/minute, and glucose <119.9 mg/dl were at increased risk with a NPAR ≥28.1.

## 4. Discussion

We demonstrated that higher NPARs were associated with an increased risk of 30-, 90-, and 365-day all-cause mortality in critically ill patients with AKI after adjustments for age, ethnicity, and gender. Furthermore, after adjusting for more confounding factors, NPAR was also an independent predictor of all-cause mortality in these patients. Moreover, NPAR was found to be a better predictor than neutrophil percentage or albumin alone. There were no significant interactions in most strata in the subgroup analyses. To our knowledge, our study is the first to find that increased NPAR was independently associated with poor prognosis in critically ill patients with AKI.

Several studies have shown that AKI was associated with local and systemic inflammatory responses [21, 22]; therefore, as markers of inflammation and immune responses, neutrophil and albumin have been shown to provide additional information regarding the prognosis of AKI [23, 24]. Combinations generating new biomarkers, including neutrophil-to-lymphocyte (NLR) and platelet-to-lymphocyte ratio (PLR), are good prognostic indicators in patients with AKI [25, 26]. Several studies have shown that hypoalbuminemia is a risk factor for the development and poor prognosis of AKI in critical illness [27–29]. Serum albumin protects the kidneys from toxic substances and maintains optimal colloid pressure to ensure renal perfusion [30]. Tawfik et al. [12] and Tingle et al. [13] suggested that NAR is an independent prognostic marker for survival in patients with solid tumors. The findings of the present study suggested that NPAR was an independent predictor of all-cause mortality in critically ill patients with AKI and was a better predictor than neutrophil percentage or albumin alone. Therefore, we have reason to believe that NPAR has important clinical significance.

AKI involves a complex physiological process caused by a series of factors, and its pathogenesis remains unclear [31]. Previous studies have proposed several possible explanations, one of which is that high circulating levels of inflammatory mediators are crucial causes of AKI. The known inflammatory mediators associated with AKI and its prognosis include neutrophils, lymphocytes, platelets, interleukin- (IL-) 6, IL-10, tumor necrosis factor receptor- (TNF-R-) I, TNF-R-II, C-reactive protein (CRP), albumin concentrations, and red blood cell distribution width (RDW) [32–34]. Another possible pathogenesis of AKI is impaired renal blood flow autoregulation [35]. Decreased renal blood flow depletes intracellular ATP, destroys intracellular calcium homeostasis, generates free radicals, activates inflammatory pathways, and destroys the integrity of the cytoskeleton [36, 37]. These lesions eventually lead to hypoxic damage to tubular cells, and damaged cells form casts obstruct renal tubules.

There were some limitations in our study. First, the study had a single-center retrospective design, and biases were inevitable. Second, we calculated NPAR only upon admission to the ICU; a single measure of NPAR may affect the accuracy of the results. Third, although we did our best to use a multivariate model to control bias, there remain numerous other known and unknown factors. Finally, retrospective of databases has many defects; therefore, multicenter, prospective studies are needed to confirm these findings.

## 5. Conclusions

We demonstrated that higher NPARs were associated with increased risk of 30-, 90-, and 365-day all-cause mortality in critically ill patients with AKI. Nevertheless, these findings need to be confirmed by large prospective multicenter studies.

## Figures and Tables

**Table 1:** Characteristics of the study patients according to neutrophil percentage-to-albumin ratios.

Characteristics	Neutrophil percentage-to-albumin ratios			*P* value
	<22.1 (*n* = 2492)	≥22.1, <28.1 (*n* = 2494)	≥28.1 (*n* = 2495)	
Age (years)	62.0 ± 18.1	65.7 ± 17.0	65.8 ± 16.6	<0.01
Gender, *n* (%)				<0.01
Female	989 (39.7)	1072 (43.0)	1184 (47.5)	
Male	1503 (60.3)	1422 (57.0)	1311 (52.5)	
Ethnicity, *n* (%)				<0.01
White	1706 (69.6)	1777 (72.4)	1741 (71.1)	
Black	331 (13.5)	231 (9.4)	221 (9.0)	
Other	414 (16.9)	448 (18.2)	486 (19.9)	
NPAR	17.4 ± 4.7	25.0 ± 1.7	35.4 ± 7.3	<0.01
SBP (mmHg)	119.8 ± 18.2	118.7 ± 17.7	114.0 ± 16.4	<0.01
DBP (mmHg)	62.9 ± 11.9	60.6 ± 11.1	58.2 ± 10.7	<0.01
MBP (mmHg)	79.6 ± 12.1	77.7 ± 11.4	75.0 ± 11.1	0.03
Heart rate (beats/minute)	86.9 ± 17.9	87.4 ± 16.9	91.1 ± 17.1	<0.01
Respiratory rate (beats/minute)	19.4 ± 4.3	19.8 ± 4.2	20.4 ± 4.6	<0.01
Temperature (°C)	36.9 ± 0.7	36.8 ± 0.7	36.8 ± 0.8	<0.01
SPO2 (%)	97.0 ± 2.8	97.0 ± 2.3	97.0 ± 3.0	0.18
Comorbidities, *n* (%)				
Coronary artery disease	360 (14.4)	524 (21.0)	384 (15.4)	<0.01
Congestive heart failure	570 (22.9)	611 (24.5)	447 (17.9)	<0.01
Atrial fibrillation	543 (21.8)	732 (29.4)	701 (28.1)	<0.01
Stroke	257 (10.3)	216 (8.7)	152 (6.1)	<0.01
Renal disease	384 (15.4)	458 (18.4)	431 (17.3)	0.02
Liver disease	203 (8.1)	259 (10.4)	339 (13.6)	<0.01
Pneumonia	701 (28.1)	827 (33.2)	922 (37.0)	0.14
Malignancy	425 (17.1)	388 (15.6)	595 (23.8)	<0.01
Respiratory failure	875 (35.1)	1015 (40.7)	1289 (51.7)	<0.01
Laboratory parameters				
Neutrophil (%)	65.8 ± 20.3	82.1 ± 8.8	85.2 ± 8.0	<0.01
Albumin (g/dl)	3.8 ± 0.6	3.3 ± 0.4	2.5 ± 0.4	<0.01
Bicarbonate (mmol/L)	20.7 ± 5.5	20.7 ± 5.5	19.6 ± 5.7	<0.01
Creatinine (mEq/L)	1.5 ± 1.7	1.7 ± 1.7	1.8 ± 1.7	<0.01
Chloride (mmol/L)	100.6 ± 6.8	101.0 ± 7.1	102.5 ± 7.7	<0.01
Glucose (mg/dl)	143.1 ± 50.0	147.1 ± 48.5	146.0 ± 52.4	<0.01
Hematocrit (%)	31.2 ± 6.5	30.1 ± 6.2	27.7 ± 5.6	<0.01
Hemoglobin (g/dl)	10.7 ± 2.3	10.2 ± 2.1	9.3 ± 1.9	<0.01
Platelet (109/L)	183.0 ± 99.5	201.1 ± 111.3	203.4 ± 139.0	<0.01
Sodium (mmol/L)	136.3 ± 5.4	136.0 ± 5.8	135.9 ± 6.2	0.01
Potassium (mmol/L)	3.7 ± 0.6	3.8 ± 0.6	3.7 ± 0.7	<0.01
BUN (mg/dl)	26.0 ± 21.7	32.7 ± 25.1	35.9 ± 26.5	<0.01
WBC (109/L)	9.7 ± 12.8	11.1 ± 6.8	12.8 ± 7.6	<0.01
PT (seconds)	14.8 ± 4.9	15.6 ± 6.1	16.0 ± 5.1	<0.01
APTT (seconds)	30.6 ± 11.8	31.5 ± 12.1	33.9 ± 12.7	<0.01
INR	1.4 ± 0.8	1.4 ± 0.7	1.5 ± 0.7	<0.001
Scoring systems				
SOFA	5.1 ± 3.6	5.5 ± 3.4	6.6 ± 3.8	<0.01
SAPSII	37.9 ± 15.5	40.7 ± 13.9	45.8 ± 15.4	<0.01
AKI stage, *n* (%)				<0.01
Stage 1	641 (25.7)	557 (22.3)	484 (19.4)	
Stage 2	386 (15.5)	413 (16.6)	436 (17.5)	
Stage 3	1465 (58.8)	1524 (61.1)	1575 (63.1)	
Renal replacement therapy, *n* (%)	214 (8.6)	253 (10.1)	322 (12.9)	<0.01
Vasopressor use, *n* (%)	841 (33.7)	921 (36.9)	1247 (50.0)	<0.01
ICU LOS (days)	4.9 ± 6.4	5.7 ± 7.2	7.1 ± 8.7	<0.01
30-day mortality, *n* (%)	386 (15.5)	510 (20.4)	797 (31.9)	<0.01
90-day mortality, *n* (%)	522 (20.9)	688 (27.6)	1032 (41.4)	<0.01
365-day mortality, *n* (%)	702 (28.2)	939 (37.7)	1283 (51.4)	<0.01

NPAR: neutrophil percentage-to-albumin ratio; SBP: systolic blood pressure; DBP: diastolic blood pressure; MBP: mean blood pressure; WBC: white blood cell; BUN: blood urea nitrogen; PT: prothrombin time; APTT: activated partial thromboplastin time; INR: international normalized ratio; SOFA: sequential organ failure assessment; SAPSII: simplified acute physiology score II; AKI: acute kidney injury; ICU: intensive care unit; LOS: length of stay.

**Table 2:** HRs (95% CIs) for all-cause mortality across groups of neutrophil percentage-to-albumin ratios.

NAR	Nonadjusted		Model I		Model II	
	HR (95% CIs)	*P* value	HR (95% CIs)	*P* value	HR (95% CIs)	*P* value
30-day all-cause mortality						
Tertiles						
<22.1	1.0 (ref)		1.0 (ref)		1.0 (ref)	
≥22.1, <28.1	1.35 (1.18, 1.54)	<0.01	1.27 (1.11, 1.45)	<0.01	1.20 (1.05, 1.38)	0.01
≥28.1	2.24 (1.99, 2.53)	<0.01	2.11 (1.86, 2.39)	<0.01	1.48 (1.30, 1.69)	<0.01
*P* trend	<0.01		<0.01		<0.01	
Quintiles						
<19.6	1.0 (ref)		1.0 (ref)		1.0 (ref)	
≥19.6, <23.2	1.04 (0.87, 1.24)	0.67	0.95 (0.79, 1.14)	0.58	0.97 (0.80, 1.17)	0.74
≥23.2, <26.7	1.29 (1.09, 1.53)	<0.01	1.18 (0.99, 1.40)	0.06	1.13 (0.94, 1.35)	0.19
≥26.7, <31.8	1.76 (1.49, 2.07)	<0.01	1.56 (1.32, 1.84)	<0.01	1.33 (1.12, 1.58)	<0.01
≥31.8	2.48 (2.13, 2.90)	<0.01	2.29 (1.96, 2.68)	<0.01	1.48 (1.25, 1.75)	<0.01
*P* trend	<0.01		<0.01		<0.01	

90-day all-cause mortality						
Tertiles						
<22.1	1.0 (ref)		1.0 (ref)		1.0 (ref)	
≥22.1, <28.1	1.36 (1.22, 1.53)	<0.01	1.28 (1.14, 1.43)	<0.01	1.21 (1.07, 1.36)	<0.01
≥28.1	2.24 (2.02, 2.49)	<0.01	2.10 (1.89, 2.34)	<0.01	1.47 (1.31, 1.66)	<0.01
*P* trend	<0.01		<0.01		<0.01	
Quintiles						
<19.6	1.0 (ref)		1.0 (ref)		1.0 (ref)	
≥19.6, <23.2	1.09 (0.93, 1.27)	0.27	1.00 (0.85, 1.16)	0.96	1.04 (0.89, 1.23)	0.62
≥23.2, <26.7	1.31 (1.13, 1.52)	<0.01	1.19 (1.02, 1.38)	0.02	1.16 (0.99, 1.35)	0.06
≥26.7, <31.8	1.84 (1.60, 2.12)	<0.01	1.63 (1.41, 1.88)	<0.01	1.39 (1.20, 1.61)	<0.01
≥31.8	2.50 (2.18, 2.86)	<0.01	2.31 (2.01, 2.65)	<0.01	1.50 (1.30, 1.74)	<0.01
*P* trend	<0.01		<0.01		<0.01	

365-day all-cause mortality						
Tertiles						
<22.1	1.0 (ref)		1.0 (ref)		1.0 (ref)	
≥22.1, <28.1	1.41 (1.28, 1.56)	<0.01	1.31 (1.18, 1.44)	<0.01	1.22 (1.10, 1.35)	<0.01
≥28.1	2.18 (1.99, 2.39)	<0.01	2.06 (1.88, 2.27)	<0.01	1.46 (1.32, 1.62)	<0.01
*P* trend	<0.01		<0.01		<0.01	
Quintiles						
<19.6	1.0 (ref)		1.0 (ref)		1.0 (ref)	
≥19.6, <23.2	1.11 (0.97, 1.27)	0.12	1.01 (0.88, 1.16)	0.87	1.06 (0.92, 1.22)	0.41
≥23.2, <26.7	1.34 (1.18, 1.52)	<0.01	1.20 (1.06, 1.37)	<0.01	1.17 (1.02, 1.33)	0.03
≥26.7, <31.8	1.83 (1.62, 2.07)	<0.01	1.61 (1.42, 1.82)	<0.01	1.36 (1.20, 1.55)	<0.01
≥31.8	2.40 (2.13, 2.70)	<0.01	2.25 (2.00, 2.54)	<0.01	1.49 (1.31, 1.70)	<0.01
*P* trend	<0.01		<0.01		<0.01	

HR: hazard ratio; CI: confidence interval. Models were derived from Cox proportional hazards regression models. Nonadjusted model, adjusted for none. Adjust I model, adjusted for age, ethnicity, and gender. Adjust II model, adjusted for age, ethnicity, gender, acute kidney injury stage, congestive heart failure, atrial fibrillation, liver disease, coronary artery disease, stroke, malignancy, respiratory failure, pneumonia, sodium, potassium, chloride, BUN, INR, APTT, platelet, WBC, hematocrit, creatinine, glucose, bicarbonate, vasopressor use, heart rate, systolic blood pressure, diastolic blood pressure, respiration rate, temperature, SPO_2_, SOFA, SAPSII, and renal replacement therapy.

**Table 3:** Subgroup analysis of the associations between the neutrophil percentage-to-albumin ratios and 30-day all-cause mortality.

	*N*	Neutrophil percentage-to-albumin ratios			*P* for interaction
		<22.1	≥22.1, <28.1	≥28.1	
CHF					0.92
No	6213	1.0 (ref)	1.28 (1.10, 1.48)	2.06 (1.81, 2.35)	
Yes	1268	1.0 (ref)	1.31 (0.93, 1.84)	2.25 (1.61, 3.14)	

AFIB					0.22
No	5505	1.0 (ref)	1.33 (1.13, 1.56)	2.22 (1.92, 2.58)	
Yes	1976	1.0 (ref)	1.08 (0.86, 1.36)	1.80 (1.45, 2.23)	

CAD					0.06
No	5853	1.0 (ref)	1.15 (0.99, 1.34)	1.90 (1.66, 2.18)	
Yes	1628	1.0 (ref)	1.70 (1.27, 2.27)	2.67 (2.00, 3.55)	

Stroke					0.22
No	6856	1.0 (ref)	1.29 (1.11, 1.49)	2.23 (1.95, 2.55)	
Yes	625	1.0 (ref)	1.23 (0.88, 1.71)	1.65 (1.18, 2.32)	

Malignancy					0.03
No	6073	1.0 (ref)	1.37 (1.17, 1.59)	2.21 (1.91, 2.55)	
Yes	1408	1.0 (ref)	1.01 (0.77, 1.31)	1.61 (1.29, 2.02)	

Liver disease					0.32
No	6680	1.0 (ref)	1.19 (1.03, 1.38)	2.01 (1.76, 2.30)	
Yes	801	1.0 (ref)	1.39 (0.98, 1.97)	1.86 (1.35, 2.57)	

Respiratory failure					<0.01
No	4302	1.0 (ref)	1.33 (1.09, 1.64)	2.48 (2.05, 3.01)	
Yes	3179	1.0 (ref)	1.10 (0.92, 1.31)	1.49 (1.27, 1.75)	

Pneumonia					0.05
No	5031	1.0 (ref)	1.38 (1.16, 1.64)	2.27 (1.94, 2.66)	
Yes	2450	1.0 (ref)	1.04 (0.84, 1.28)	1.71 (1.41, 2.07)	

AKI stage					0.02
Stage 1	1682	1.0 (ref)	1.11 (0.83, 1.48)	2.36 (1.82, 3.08)	
Stage 2	1235	1.0 (ref)	1.08 (0.77, 1.51)	1.44 (1.05, 1.98)	
Stage 3	4564	1.0 (ref)	1.35 (1.14, 1.59)	2.16 (1.85, 2.52)	

WBC (109/L)					0.07
<7.6	2449	1.0 (ref)	1.19 (0.95, 1.49)	1.85 (1.50, 2.29)	
≥7.6, <12.1	2528	1.0 (ref)	1.58 (1.23, 2.03)	2.53 (1.98, 3.22)	
≥12.1	2502	1.0 (ref)	0.98 (0.78, 1.23)	1.61 (1.31, 1.99)	

Sodium (mmol/L)					0.09
<134	2010	1.0 (ref)	1.05 (0.82, 1.35)	1.85 (1.48, 2.30)	
≥134, <138	2333	1.0 (ref)	1.40 (1.11, 1.76)	1.87 (1.49, 2.34)	
≥138	3136	1.0 (ref)	1.27 (1.02, 1.57)	2.34 (1.92, 2.85)	

BUN (mg/dl)					0.28
<17	2295	1.0 (ref)	1.45 (1.09, 1.94)	1.90 (1.44, 2.52)	
≥17, <34	2677	1.0 (ref)	1.19 (0.96, 1.48)	2.06 (1.68, 2.52)	
≥34	2506	1.0 (ref)	1.02 (0.83, 1.26)	1.68 (1.39, 2.03)	

INR					0.06
<1.2	2404	1.0 (ref)	1.36 (1.05, 1.76)	2.52 (1.96, 3.24)	
≥1.2, <1.4	2176	1.0 (ref)	1.06 (0.81, 1.39)	1.59 (1.24, 2.05)	
≥1.4	2639	1.0 (ref)	1.11 (0.91, 1.35)	1.62 (1.36, 1.93)	

Potassium (mmol/L)					<0.01
<3.5	2415	1.0 (ref)	1.06 (0.83, 1.34)	1.52 (1.23, 1.88)	
≥3.5, <4	2560	1.0 (ref)	1.23 (0.97, 1.56)	2.24 (1.80, 2.79)	
≥4	2505	1.0 (ref)	1.40 (1.12, 1.75)	2.49 (2.02, 3.06)	

APTT (seconds)					0.48
<26.7	2402	1.0 (ref)	1.29 (1.00, 1.67)	2.11 (1.63, 2.72)	
≥26.7, <32.2	2404	1.0 (ref)	1.29 (1.01, 1.66)	1.91 (1.52, 2.41)	
≥32.2	2405	1.0 (ref)	1.05 (0.85, 1.28)	1.65 (1.37, 1.98)	

Platelet (109/L)					0.01
<139	2468	1.0 (ref)	1.10 (0.90, 1.35)	1.61 (1.35, 1.93)	
≥139, <223	2492	1.0 (ref)	1.56 (1.23, 1.99)	2.39 (1.89, 3.03)	
≥223	2518	1.0 (ref)	1.30 (1.00, 1.71)	2.47 (1.93, 3.15)	

Hematocrit (%)					<0.01
<26.9	2470	1.0 (ref)	0.88 (0.69, 1.12)	1.60 (1.31, 1.95)	
≥26.9, <32.3	2515	1.0 (ref)	1.29 (1.02, 1.63)	1.85 (1.48, 2.31)	
≥32.3	2495	1.0 (ref)	1.57 (1.26, 1.96)	3.05 (2.44, 3.81)	

Creatinine (mEq/L)					0.13
<0.9	2440	1.0 (ref)	1.59 (1.22, 2.09)	2.41 (1.88, 3.10)	
≥0.9, <1.5	2490	1.0 (ref)	1.21 (0.96, 1.52)	2.03 (1.62, 2.53)	
≥1.5	2549	1.0 (ref)	1.00 (0.82, 1.22)	1.67 (1.39, 2.00)	

Bicarbonate (mg/dl)					<0.01
<18	2068	1.0 (ref)	1.00 (0.81, 1.24)	1.38 (1.14, 1.68)	
≥18, <23	2775	1.0 (ref)	1.24 (0.99, 1.57)	2.08 (1.68, 2.58)	
≥23	2636	1.0 (ref)	1.51 (1.18, 1.94)	2.72 (2.15, 3.45)	

Glucose (mg/dl)					0.03
<119.9	2485	1.0 (ref)	1.43 (1.11, 1.84)	2.63 (2.10, 3.30)	
≥119.9, <152.8	2484	1.0 (ref)	1.39 (1.10, 1.76)	2.16 (1.73, 2.71)	
≥152.8	2485	1.0 (ref)	1.00 (0.82, 1.24)	1.62 (1.33, 1.96)	

Chloride (mmol/L)					0.76
<99	2206	1.0 (ref)	1.17 (0.94, 1.47)	1.90 (1.53, 2.35)	
≥99, <104	2370	1.0 (ref)	1.35 (1.07, 1.70)	2.33 (1.87, 2.89)	
≥104	2903	1.0 (ref)	1.24 (0.98, 1.58)	2.12 (1.72, 2.63)	

SBP (mmHg)					0.02
<108	2486	1.0 (ref)	0.99 (0.81, 1.21)	1.53 (1.28, 1.83)	
≥108, <123	2485	1.0 (ref)	1.47 (1.15, 1.88)	2.21 (1.75, 2.79)	
≥123	2486	1.0 (ref)	1.40 (1.08, 1.81)	2.40 (1.87, 3.07)	

DBP (mmHg)					0.36
<55	2485	1.0 (ref)	1.19 (0.96, 1.48)	2.01 (1.65, 2.44)	
≥55, <64	2486	1.0 (ref)	1.07 (0.85, 1.35)	1.81 (1.47, 2.23)	
≥64	2486	1.0 (ref)	1.50 (1.18, 1.92)	2.22 (1.74, 2.83)	

Heart rate (beats/minute)					0.02
<80	2487	1.0 (ref)	1.52 (1.20, 1.92)	2.63 (2.10, 3.31)	
≥80, <96	2488	1.0 (ref)	1.26 (0.99, 1.61)	2.04 (1.63, 2.55)	
≥96	2490	1.0 (ref)	1.00 (0.80, 1.24)	1.54 (1.27, 1.86)	

Respiratory rate (beats/minute)					<0.01
<18	2483	1.0 (ref)	1.72 (1.31, 2.25)	2.62 (2.03, 3.38)	
≥18, <22	2482	1.0 (ref)	1.28 (1.01, 1.64)	2.45 (1.96, 3.06)	
≥22	2485	1.0 (ref)	0.93 (0.76, 1.13)	1.40 (1.17, 1.67)	

Temperature (°C)					0.04
<36.5	2464	1.0 (ref)	1.47 (1.19, 1.83)	2.37 (1.94, 2.88)	
≥36.5, <37.1	2465	1.0 (ref)	1.28 (1.00, 1.66)	2.37 (1.87, 2.99)	
≥37.1	2470	1.0 (ref)	1.04 (0.83, 1.32)	1.55 (1.25, 1.93)	

SPO2 (%)					0.07
<96.5	2478	1.0 (ref)	1.06 (0.86, 1.30)	2.01 (1.67, 2.43)	
≥96.5, <98.3	2491	1.0 (ref)	1.17 (0.91, 1.51)	2.05 (1.63, 2.57)	
≥98.3	2485	1.0 (ref)	1.61 (1.26, 2.05)	2.28 (1.81, 2.87)	

SOFA score					<0.01
<4	2295	1.0 (ref)	1.49 (1.09, 2.05)	2.52 (1.83, 3.45)	
≥4, <7	2581	1.0 (ref)	1.24 (0.96, 1.59)	2.01 (1.59, 2.54)	
≥7	2605	1.0 (ref)	1.02 (0.85, 1.22)	1.34 (1.14, 1.58)	

SAPSII score					<0.01
<34	2375	1.0 (ref)	1.42 (0.93, 2.16)	2.93 (1.97, 4.36)	
≥34, <47	2607	1.0 (ref)	1.36 (1.06, 1.74)	1.98 (1.56, 2.52)	
≥47	2499	1.0 (ref)	1.02 (0.86, 1.21)	1.27 (1.09, 1.48)	

Vasopressor use					<0.01
No	4472	1.0 (ref)	1.48 (1.20, 1.83)	2.66 (2.18, 3.25)	
Yes	3009	1.0 (ref)	1.03 (0.86, 1.22)	1.40 (1.20, 1.64)	

Renal replacement therapy					<0.01
No	6692	1.0 (ref)	1.24 (1.07, 1.43)	2.17 (1.90, 2.48)	
Yes	789	1.0 (ref)	1.16 (0.83, 1.63)	1.34 (0.97, 1.84)	

CHF: congestive heart failure; AFIB: atrial fibrillation; CAD: coronary artery disease; AKI: acute kidney injury; WBC: white blood cell; BUN: blood urea nitrogen; INR: international normalized ratio; APTT: activated partial thromboplastin time; SBP: systolic blood pressure; DBP: diastolic blood pressure; SOFA: sequential organ failure assessment; SAPSII: simplified acute physiology score II.

## Data Availability

The clinical data used to support the findings of this study were supplied by Monitoring in Intensive Care Database III version 1.4 (MIMIC-III v.1.4). Although the database is publicly and freely available, researchers must complete the National Institutes of Health's web-based course known as Protecting Human Research Participants to apply for permission to access the database.

## Supplementary Materials

Supplementary Materials The occurrence of AKI was determined on the basis of Kidney Disease: Improving Global Outcomes (KDIGO) definition [17], and Structured Query Language (SQL) for extracting AKI was included in supplementary material.
