# Supplementary material for: The Neutrophil Percentage-to-Albumin Ratio Is Associated with All-Cause Mortality in Critically Ill Patients with Acute Kidney Injury
Source: Biomed Res Int. 2020 Feb 18;2020:5687672. doi: 10.1155/2020/5687672 (PMC7049452; doi:10.1155/2020/5687672)
Supplement: Supplementary Materials — The occurrence of AKI was determined on the basis of Kidney Disease: Improving Global Outcomes (KDIGO) definition [17], and Structured Query Language (SQL) for extracting AKI was included in supplementary material. [file 5687672.f1.pdf]

```
-- This query checks if the patient had AKI according to KDIGO.
-- AKI is calculated every time a creatinine or urine output measurement occurs.
-- Baseline creatinine is defined as the lowest creatinine in the past 7 days.


DROP MATERIALIZED VIEW IF EXISTS kdigo_stages CASCADE;
CREATE MATERIALIZED VIEW kdigo_stages AS
-- get creatinine stages
with cr_stg AS
(
  SELECT
    cr.icustay_id
    , cr.charttime
    , cr.creat
    , case
        -- 3x baseline
        when cr.creat >= (cr.creat_low_past_7day*3.0) then 3
        -- *OR* cr >= 4.0 with associated increase
        when cr.creat >= 4
        -- For patients reaching Stage 3 by SCr >4.0 mg/dl
        -- require that the patient first achieve ... acute increase >= 0.3 within 48 hr
        -- *or* an increase of >= 1.5 times baseline
        and (cr.creat_low_past_48hr <= 3.7 OR cr.creat >=
(1.5*cr.creat_low_past_7day))
            then 3
        -- TODO: initiation of RRT
        when cr.creat >= (cr.creat_low_past_7day*2.0) then 2
        when cr.creat >= (cr.creat_low_past_48hr+0.3) then 1
        when cr.creat >= (cr.creat_low_past_7day*1.5) then 1
    else 0 end as aki_stage_creat
  FROM kdigo_creat cr
)
-- stages for UO / creat
, uo_stg as
```

```
(
  select
        uo.icustay_id
      , uo.charttime
      , uo.weight
      , uo.uo_rt_6hr
      , uo.uo_rt_12hr
      , uo.uo_rt_24hr
      -- AKI stages according to urine output
      , CASE
          WHEN uo.uo_rt_6hr IS NULL THEN NULL
          -- require patient to be in ICU for at least 6 hours to stage UO
          WHEN uo.charttime <= ie.intime + interval '6' hour THEN 0
          -- require the UO rate to be calculated over half the period
          -- i.e. for uo rate over 24 hours, require documentation at least 12 hr apart
          WHEN uo.uo_tm_24hr >= 11 AND uo.uo_rt_24hr < 0.3 THEN 3
          WHEN uo.uo_tm_12hr >= 5 AND uo.uo_rt_12hr = 0 THEN 3
          WHEN uo.uo_tm_12hr >= 5 AND uo.uo_rt_12hr < 0.5 THEN 2
          WHEN uo.uo_tm_6hr >= 2 AND uo.uo_rt_6hr    < 0.5 THEN 1
      ELSE 0 END AS aki_stage_uo
  from kdigo_uo uo
  INNER JOIN icustays ie
      ON uo.icustay_id = ie.icustay_id
)
-- get all charttimes documented
, tm_stg AS
(
      SELECT
        icustay_id, charttime
      FROM cr_stg
      UNION
      SELECT
        icustay_id, charttime
      FROM uo_stg
)
select
      ie.icustay_id
    , tm.charttime
    , cr.creat
    , cr.aki_stage_creat
    , uo.uo_rt_6hr
    , uo.uo_rt_12hr
    , uo.uo_rt_24hr
    , uo.aki_stage_uo
```

```sql
    -- Classify AKI using both creatinine/urine output criteria
    , GREATEST(cr.aki_stage_creat, uo.aki_stage_uo) AS aki_stage
FROM icustays ie
-- get all possible charttimes as listed in tm_stg
LEFT JOIN tm_stg tm
    ON ie.icustay_id = tm.icustay_id
LEFT JOIN cr_stg cr
    ON ie.icustay_id = cr.icustay_id
    AND tm.charttime = cr.charttime
LEFT JOIN uo_stg uo
    ON ie.icustay_id = uo.icustay_id
    AND tm.charttime = uo.charttime
order by ie.icustay_id, tm.charttime;
```
